# Financial Outcomes After Traumatic Injury Among Working-Age US Adults With Commercial Insurance

**DOI:** 10.1001/jamahealthforum.2022.4105

**Published:** 2022-11-11

**Authors:** John W. Scott, Kirstin W. Scott, Michelle Moniz, Erin F. Carlton, Renuka Tipirneni, Nora Becker

**Affiliations:** 1 Center for Healthcare Outcomes and Policy, Department of Surgery, University of Michigan, Ann Arbor; 2 Department of Emergency Medicine, University of Michigan, Ann Arbor; 3 Department of Obstetrics and Gynecology, University of Michigan, Ann Arbor; 4 Division of Pediatric Critical Care Medicine, Department of Pediatrics, University of Michigan, Ann Arbor; 5 Division of General Medicine, Department of Internal Medicine, University of Michigan, Ann Arbor

## Abstract

This cross-sectional study links insurance claims and consumer credit report data to evaluate the experience of financial distress in commercially insured adults after traumatic injury.

## Introduction

One in 5 US households held medical debt in 2021, totaling $88 billion.^1^ Health shocks, such as traumatic injury, pose significant risk to the health and financial well-being of working-age adults. The degree to which commercially insured patients hospitalized for traumatic injuries experience financial distress remains unknown.

## Methods

This cross-sectional study links Blue Cross Blue Shield of Michigan preferred provider organization insurance claims from 2019 through 2021 (exclusive of July-December 2020) to January 2021 Experian consumer credit reports. We identified working-age adults (aged 21-64 years) whose January 2021 credit reports occurred more than 6 months after hospital admission for traumatic injury (between January 2019 and June 2020). We used coarsened exact matching of age, sex, calendar quarter of admission, and zip code social vulnerability index to identify comparison patients admitted between January and December 2021 for traumatic injuries after their January 2021 credit report. Race and ethnicity were not included because the claims database did not reliably collect these data. The University of Michigan institutional review board approved this study, and deemed waived the requirement for informed consent because deidentified data were used. This study followed the STROBE reporting guideline.

Financial outcomes included any delinquent debt, debt in collections (medical and nonmedical), bankruptcy filings in the past 2 years, and credit score. Multivariable logistic and linear regression models incorporated matching weights to compare financial outcomes between the cohorts. A 2-sided *P* < .05 was considered significant. Analyses were performed using Stata, version 17.0 (StataCorp LLC) software. The eMethods in the Supplement provide additional detail.

## Results

The 3165 working-age adults in the postinjury cohort were similar demographically to the 2223 patients in the comparison cohort (Table 1). Relative to the comparison cohort, the postinjury cohort had a 23% higher likelihood of having medical debt in collections (754 [23.8%] vs 429 [19.3%]; *P* < .001), a 70% higher amount of medical debt in collections (mean [SD], $2087 [$6235] vs $1227 [$1227]; *P* < .001), and a 110% higher bankruptcy rate (39 [1.2%] vs 13 [0.6%]; *P* = .03) (Table 2). No significant differences were found regarding credit scores or amount of nonmedical debt in collections.

**Table 1.** Characteristics of the Postinjury and Matched Comparison Cohorts^a^

Characteristic	Weighted %		*P* value
	Postinjury cohort (n = 3165)	Comparison cohort (n = 2223)	
Age (y), mean (SD)	48.5 (13.4)	48.4 (13.4)	.84
Age categories, y			
21-24	8.2	8.2	>.99
25-34	12.3	12.3	>.99
35-44	13.1	13.1	>.99
45-54	21.1	21.1	>.99
55-64	45.4	45.4	>.99
Sex			
Female	41.4	41.4	>.99
Male	58.6	58.6	
Length of stay, mean (SD), d	5.0 (4.9)	5.1 (4.8)	.40
Length of stay categories, d			
<3	37.4	34.5	.04
4-7	39.7	41.7	.17
>7	22.9	23.8	.47
Intensive care use, any	38.3	37.2	.42
Calendar quarter of admission			
January-March	30.6	30.6	>.99
April-June	34.4	34.4	>.99
July-September	20.4	20.4	>.99
October-December	14.5	14.5	>.99
SVI, overall score^b^	0.45	0.45	.82
SVI subscores, mean (SD)			
Socioeconomic status	0.43 (0.22)	0.43 (0.22)	.83
Household composition	0.47 (0.20)	0.47 (0.20)	.97
Minority status/language	0.46 (0.20)	0.47 (0.20)	.30
Housing and transportation	0.49 (0.18)	0.49 (0.18)	.67
Distribution of SVI quartiles^c^			
First, least vulnerable (0.00-0.25)	19.3	19.3	>.99
Second (0.25-0.49)	38.4	38.4	>.99
Third (0.50-0.74)	33.8	33.8	>.99
Fourth, most vulnerable (0.75-1.00)	8.5	8.5	>.99

Abbreviation: SVI, social vulnerability index.

^a^ Both the postinjury and matched comparison cohorts had credit data obtained in January 2021. The postinjury cohort consists of patients whose January 2021 credit data were obtained 7 to 24 months after they were admitted for injury. The comparison cohort consists of patients whose January 2021 credit data were obtained before they were ever admitted for an injury. The matched comparison cohort was created using coarsened exact matching based on age, sex, admission quarter, and zip code SVI.

^b^ SVI ranges from 0 to 1.

^c^ Among the 2046 injured patients and 1426 matched patients with in-state zip codes available.

**Table 2.** Financial Outcomes After Hospital Admission for Traumatic Injury in the Postinjury vs Matched Comparison Cohorts^a^

Outcome	Postinjury cohort	Comparison cohort	Adjusted difference^b^	Relative change, %	*P* value
Sample size, No.	3165	2223	NA	NA	NA
Credit score outcomes					
Average credit score, mean (SD)	705 (103)	702 (101)	2.6	0.4	.38
Subprime credit score (≤600), No. (%)	635 (20.1)	437 (19.8)	0.4^c^	1.8	.76
Any bankruptcy in past 2 y, No. (%)	39 (1.2)	13 (0.6)	0.7^c^	110	.03
Any debt in collections, No. (%)					
Medical	754 (23.8)	429 (19.3)	4.5^c^	23	<.001
Nonmedical	490 (15.5)	358 (16.1)	−0.6^c^	−4	.57
Amount of debt in collections, $^d^					
Medical					
Mean (SD)	2087 (6235)	1227 (2109)	860	70	.001
Median (IQR)	731 (309-1816)	540 (233-1287)	191	35	.005
Nonmedical					
Mean (SD)	2358 (4150)	2075 (3379)	283	14	.29
Median (IQR)	976 (400-2451)	983 (398-2289)	−7	−0.7	.96

Abbreviation: NA, not applicable.

^a^ Both the postinjury and matched comparison cohorts had credit data obtained in January 2021. The postinjury cohort consists of patients whose January 2021 credit data were obtained 7 to 24 months after they were admitted for injury. The comparison cohort consists of patients whose January 2021 credit data were obtained before they were ever admitted for an injury. The comparison cohort was created using coarsened exact matching based on age, sex, admission quarter, and zip code–based social vulnerability index.

^b^ Adjusted differences account for matching weights, and SEs are clustered by zip code.

^c^ Adjusted differences correspond to the percentages in parentheses for the postinjury and comparison cohorts.

^d^ Among patients with any debt in collections.

## Discussion

Admission for traumatic injury was associated with higher rates and amounts of medical debt in collections and a doubling of bankruptcy filings among commercially insured working-age adults. No difference was found in credit scores or nonmedical debt in collections. Consistent with previous work,^2^ we found that for many commercially insured patients, the burden of out-of-pocket (OOP) costs after hospitalization exceeds their ability to pay and could be associated with bankruptcy risk. Another study of commercially insured adults found that the average OOP payments in the year after traumatic injury was $3400 overall and approximately $5000 for patients with high deductibles.^3^ An association has been noted between financial distress and worse long-term physical and mental health after injury^4^; strategies to reduce financial distress may improve financial and clinical well-being.

Study limitations include the inability to adjust for other patient traits associated with financial outcomes (eg, comorbidities, wealth, income loss) and generalizability to publicly insured or uninsured patients. A cross-sectional analysis cannot rule out reverse causality (ie, potential for increased risk of injury due to significant medical debt), but the lower amount of medical debt in our comparison cohort who became injured suggests this is unlikely. Longitudinal data before and after injury are needed to better understand this association.

One policy solution to mitigate financial distress after health shocks is to reduce or eliminate cost sharing for conditions not subject to overuse, such as traumatic injury requiring prolonged hospitalization. Another alternative is to establish means-based deductibles,^5^ which could reduce the burden of OOP spending relative to income so that patients are not forced to choose between delaying care and defaulting on debt. State, community, or hospital programs that reduce financial barriers to postdischarge care may help mitigate affordability-induced delays to obtaining clinical care needed for optimal long-term recovery.^6^

## Notes

Supplement.
eMethods
