# Supplementary material for: Financial Outcomes After Traumatic Injury Among Working-Age US Adults With Commercial Insurance
Source: JAMA Health Forum. 2022 Nov 11;3(11):e224105. doi: 10.1001/jamahealthforum.2022.4105 (PMC9652745; doi:10.1001/jamahealthforum.2022.4105)
Supplement: Supplement. — eMethods [file jamahealthforum-e224105-s001.pdf]

Supplemental Online Content

Scott JW, Scott KW, Moniz M, Carlton EF, Tipirneni R, Becker N. Financial outcomes after traumatic injury among working-age US adults with commercial insurance. *JAMA Health Forum*. 2022;3(11):e224105. doi:10.1001/jamahealthforum.2022.4105

eMethods.

This supplemental material has been provided by the authors to give readers additional information about their work.

© 2022 Scott JW et al. *JAMA Health Forum.*

eMethods

Defining patient cohorts and coarsened exact matching:

Since this is a cross-sectional study, we do not have financial outcomes of interest for patients before and after hospitalization of traumatic injury. Instead, we have credit data obtained for all patients enrolled in a preferred provider organization plan offered by Blue Cross Blue Shield of Michigan in January 2021. This cross-section of credit data (from January 2021) were linked to 3 years of claims data ranging from January 2019 through December 2021. For patients who were hospitalized for a traumatic injury between January 2021 and December 2021, their credit data (from January 2021) was obtained before their injury. Conversely, for patients who were hospitalized for a traumatic injury between January 2019 and December 2020, their credit data (from January 2021) was obtained after their injury. Prior to July 2022, the three main credit bureaus allowed for a 6-month grace period between the time that medical debt was sent to collections and the time that it appears on consumer credit reports. Although the time interval between injury and unpaid bills being sent to collections agencies varies, we elected to use a 6-month washout period (July 2020-December 2020) during which injury-related medical bills would not be expected to impact credit reports.

As such, our post-injury cohort was made up of 18-64 year-old individuals (working-age adults) admitted to a hospital between January 2019 and June 2020 (thus their credit data was obtained "post-injury"). In order to evaluate their financial data in comparison to a relevant control group, we elected to draw from a population of patients who would later go on to be hospitalized for traumatic injury between January 2021 and December 2021 (i.e. those whose credit data was obtained before their hospitalization). We then used coarsened exact matching to create a matched comparison cohort based on age (groups: 21-24, 25-34, 35-44, 45-54, 55-64 years), sex (male, female), calendar quarter of admission (Jan-Mar, Apr-Jun, Jul-Sep, Oct-Dec), and quartile of social vulnerability index (SVI) (0.0-0.24, 0.25-0.49, 0.50-0.74, 0.75-0.99, missing). SVI was based on ZIP code and patients without a valid Michigan ZIP code were

© 2022 Scott JW et al. *JAMA Health Forum.*

grouped as a single zip code category. ZIP codes were invalid or missing for 35.3% of comparison cohort and 35.3% of post-injury cohort after matching (p = 1.000).

Additional detail regarding credit outcomes:

All credit outcomes were obtained from the January 2021 credit reports from the Experian consumer credit agency. ***Medical debt in collections*** and ***non-medical debt in collections*** refers to debt that has been sold to a third-party collections agency, which is typically done when debt is more than 1 to 6 months past due. Prior to July 2022, medical debt sent to collections was not reported on a consumer credit report for a 6-month grace period. Experian uses the VantageScore 4.0 ***credit score***, which is a proprietary credit score that ranges from 300 to 850. The VantageScore 4.0 is often categorized as *superprime* (781 to 850), *prime* (661 to 780), *near prime* (601 to 660), and *subprime* (300 to 600). In this analysis we defined patients with a credit score of 600 or less as having a ***subprime credit score*** because a poor credit score of 600 or lower is associated with difficulty obtaining a competitive interest rate, difficulty opening a new line of credit, and (in some states) difficulty securing employment. Lastly, Experian also reports the number of times that patients have filed for ***bankruptcy*** in the previous 24 months. For all outcome analyses, we used linear, logistic, or median regression that accounted for matching weights and we clustered standard errors at the ZIP code level.

Additional Information:

This study represents secondary use of previously obtained claims data. The deidentified database available to our research team did not reliably include information on race and ethnicity and so these variables were not used in this study.

© 2022 Scott JW et al. *JAMA Health Forum.*
